# From Clinical Trial Awareness to Practice: Factors Associated with Intent to Omit Axillary Surgery Based on the SOUND Clinical Trial

**DOI:** 10.1245/s10434-026-19792-x

**Published:** 2026-05-14

**Authors:** Ko Un Park, Megan Delisle, Michael J. Hassett, Christina A. Minami, Rinaa S. Punglia, Samuel R. Woodbury, Mary Brindle, Elizabeth A. Mittendorf, Tari A. King

**Affiliations:** 1 Division of Breast Surgery, Department of Surgery, Brigham and Women’s Hospital, Dana-Farber Cancer Institute, Boston, MA USA; 2 Ariadne Labs, Brigham and Women’s Hospital, Harvard T.H. Chan School of Public Health, Boston, MA USA; 3 Breast Oncology Program, Dana-Farber Brigham Cancer Center, Boston, MA USA; 4 Harvard Medical School, Boston, MA USA; 5 Department of Surgery, University of Manitoba, Winnipeg, MB Canada; 6 Medical Oncology, Dana-Farber Cancer Institute, Boston, MA USA; 7 Present Address: Department of Surgery, Cumming School of Medicine, University of Calgary, Alberta, Canada; 8 Present Address: Department of Oncology, Cumming School of Medicine, University of Calgary, Calgary, Canada; 9 Department of Radiation Oncology, Brigham and Women’s Hospital, Boston, MA USA; 10 Present Address: Division of Breast Surgery, Department of Surgery, Beth Israel Deaconess Medical Center, Boston, MA USA; 11 Present Address: Winship Cancer Institute of Emory University, Atlanta, GA USA

**Keywords:** Sentinel lymph node biopsy, Axillary surgery, Breast cancer, Treatment de-escalation, De-implementation

## Abstract

**Background:**

The SOUND trial, published in 2023, demonstrated the oncologic safety of sentinel lymph node biopsy (SLNB) for select patients with early-stage breast cancer. We evaluated whether earlier awareness of the results of the SOUND trial was associated with increased intent to implement SLNB omission.

**Methods:**

This multicenter, cross-sectional study surveyed North American physicians on current SLNB use, awareness of the SOUND trial results, and intent to implement trial findings. Intent was measured using the Innovation-Specific Implementation Intentions scale and scenario-based questions (ages 50 vs 60; whole- vs partial-breast irradiation), on a 5-point scale. The primary outcome was high intent to omit SLNB (“moderate” to “very great” extent). Multiple logistic regression identified factors associated with intent.

**Results:**

The response rate was 21% (208/1004): 148 (71.5%) surgeons, 32 (13.04%) medical and 27 (15.46%) radiation oncologists. Overall, 47.8% had already omitted SLNB in select patients aged < 70 years, and 63.6% reported high intent to use SOUND results. Among those who had not yet omitted SLNB (*n* = 108), 59.3% had high intent. Length of awareness of the SOUND trial (odds ratio 1.13) and practicing in Northeast USA vs Western USA (odds ratio 3.45, *p* = 0.032) were associated with increased intent to omit SLNB. In scenario-based questions, most endorsed omission for a 60-year-old patient receiving whole-breast irradiation (*n* = 105 [50.5%]); willingness decreased for a 50 year old or when partial-breast irradiation was planned. Across scenarios, intent was not associated with SOUND awareness duration, clinician characteristics, or referral workflows.

**Conclusions:**

Earlier awareness of the SOUND trial and regional differences were associated with greater intent to omit SLNB.

**Supplementary Information:**

The online version contains supplementary material available at 10.1245/s10434-026-19792-x.

## Introduction

Sentinel lymph node biopsy (SLNB) has been the standard approach to axillary staging in early-stage breast cancer for decades. The SOUND trial demonstrated that, among women with clinically node-negative, early-stage breast cancer and a negative axillary ultrasound, omission of SLNB does not compromise 5-year distant disease-free survival.^1^ The oncologic safety of SLNB omission has since been confirmed by additional international trials, including INSEMA and BOOG 2013-08, and adopted into the American Society of Clinical Oncology guideline supporting de-escalation of axillary surgery in carefully selected patients.^2–4^

Implementing SLNB omission in routine practice is complex. Axillary staging may influence decisions about radiation and systemic therapy. As a result, translating trial evidence into real-world practice requires alignment among surgeons, medical oncologists, and radiation oncologists and may be influenced by local practice culture and regional norms. This was particularly evident in the implementation of omission of SLNB in patients aged ≥ 70 years, per the 2016 Choosing Wisely statement.^5^ Despite the Choosing Wisely statement recommending avoidance of routine use of SLNB in patients aged ≥ 70 years with early-stage breast cancer undergoing lumpectomy, SLNB continues to be performed in up to 87% of these older patients for whom SLNB omission is recommended.^6–8^

The SOUND trial concept was first published in 2012, with subsequent reports on trial design and arm function outcomes in 2015–2016.^9,10^ However, the primary oncologic results were not presented until 2021 and published in 2023, more than a decade after the initial concept.^1^ This extended interval raises the important question of whether earlier awareness of a de-escalation trial—through concept papers, conference presentations, or professional networks—facilitates earlier acceptance and adoption of trial results that support practice change.

Implementation frameworks propose that implementation is a multileveled, multiphase process that involves policies, organizations, and individual providers or clinicians. Conceptual models of dissemination and implementation, such as the framework described by Baumann et al.^11^ describe a dissemination process for new knowledge that starts with knowledge inquiry, followed by knowledge synthesis, communication, interaction, persuading, activation, and research transfer. Within this framework, early exposure to trial concepts could move clinicians further along the pathway toward activation (clinician starts to act based on the new knowledge) and implementation in response to promising trial concepts and results presented at scientific meetings, even before full peer‑reviewed publications are available. Furthermore, before implementation, it is critical to understand clinicians’ attitudes toward the selected innovation and their intentions to use it.^12,13^ Studies have shown a positive relationship between intent and behavior of clinicians.^14^

Thus, we aimed to understand through a multicenter, cross-sectional survey of North American surgeons, medical oncologists, and radiation oncologists how awareness timing relates to practice patterns and intent to change practice based on the SOUND clinical trial. Specifically, we evaluated (1) whether longer awareness of SOUND was associated with discussing or implementing SLNB omission in patients aged < 70 years; (2) how intent to use SOUND results varied by clinician characteristics, practice setting, and region; and (3) how clinicians applied SLNB omission in standardized clinical scenarios.

## Methods

### Survey Development

We developed the SOUND trial dissemination survey to evaluate physicians’ awareness of the SOUND clinical trial and to assess their intent to change clinical practice based on the trial results (Supplementary file). We followed a multi-step survey development process that included literature review, interviews with subject-matter experts, conceptualization of construct and item generation, expert validation, and cognitive pretesting.^15–17^ After refining the items, cognitive pretesting was performed with five breast cancer providers. They were asked to restate each item to ensure the respondent interpretation was as designed. No items were deemed redundant or missing from the survey. Based on the user pre-testing, the final survey included 18 items in addition to the demographic information.

The key concepts we aimed to test were when and how respondents first became aware of the SOUND trial. The survey covered three different components to understand the current practice with SLNB omission and intent to change future practice. First, we ascertained current practice regarding SLNB omission in patients aged < 70 and ≥ 70 years. Next, we used a validated three-item measurement called the Innovation-Specific Implementation Intentions (MISII) questionnaire.^18^ This questionnaire covers three aspects of intentions: plans, desires, and scope. Lastly, we asked participants to respond to four scenario-based questions. The MISII and scenario-based questions were answered on a 5-point Likert scale. The verb-tense in MISII questions (e.g., current use vs planned future use of SOUND trial results) were adjusted based on respondents current practice pattern with respect to SLNB omission in patients aged < 70 years. Each provider answered only one version of the MISII questions, depending on their earlier responses. The MISII questions were as follows: (1) I use/plan to use the results from the SOUND clinical trial to guide my clinical decision-making; (2) Using the results from the SOUND clinical trial in my clinical decision-making is a high priority for me; (3) I use/will use all aspects of SOUND clinical trial results in my clinical decision-making with my patients.

### Survey Respondents

We used a nonprobability sampling strategy consisting of (1) an initial purposive sample and (2) subsequent snowball/chain-referral sampling. For the initial purposive sample, the study team compiled a list of breast cancer providers practicing in the USA and Canada using the study team’s professional networks and institutional breast program directories. Eligible participants were physicians in breast medical oncology, breast surgical oncology, or radiation oncology who were actively involved in breast cancer management in the USA or Canada. Invitees were selected based on their specialties and geographical representation. The initial invitation list was assembled intentionally to obtain representation across physician specialty and geography, including both US and Canadian respondents and a range of practice regions. No formal sampling quotas were prespecified.

We administered the survey electronically (REDCap) from August to December 2024 (US respondents) and March to May 2025 (Canadian respondents). Potential respondents received up to three email reminders to complete the survey during the data collection period.^19^ No incentives were offered. Additional participation was solicited through snowball and chain referral sampling by asking the participants to forward the survey link to others who met the inclusion criteria.^20^ For example, when the medical oncologist of a practice was sent the survey link, we asked that physician to forward the survey link to their breast medical oncologists, surgeons, and radiation oncologists and let us know the number of potential respondents to whom the survey link was forwarded. Although data on the total number of survey links forwarded were consistently collected, we did not consistently receive information on forwarding by specialty of the physicians. Thus, this estimation method may not capture the full complexity of the response rate because of variability in how participants disseminated the survey link. Repeated submissions were limited through use of CAPTCHA. Institutional review board approval was obtained (2024P001543), but the timing of the approval meant the Canadian survey was distributed after the US survey. Respondent race and ethnicity was not collected to help assure the anonymity of the respondents.

### Statistical Analysis

Simple description and comparison of means were analyzed from survey data. The outcome of interest was intent to omit SLNB to a “high extent,” defined as survey answers of “moderate” to “very great extent” on a 5-point Likert scale. In both the MISII questions and the scenario-based questions, propensity for omitting SLNB was analyzed by respondent characteristics, including length of awareness of the SOUND trial, years in practice, specialty (breast surgical oncologist with or without fellowship training, medical oncologist, or radiation oncologist), proportion of clinical practice in breast (100% in breast vs not), region of practice (Northeast, Midwest, South, West, and Canada), practice type (academic medical center vs non-academic center), frequency that respondent discusses enrollment in clinical trials with patients (often/always or not), and medical oncology/radiation oncology referral workflows (e.g. before surgery vs after surgery vs other). The patterns of missing data were assessed to ensure they were random and did not systematically affect our findings, and consistent rules for item non-response were used to handle missing items.

Separate multiple logistic regression models were applied to assess the association between respondent characteristics and each outcome of interest. All characteristics were simultaneously entered into the models as covariates to estimate adjusted odds ratios (ORs) and 95% confidence intervals (CIs) for each factor in relation to each outcome. Model assumptions were assessed, and statistical significance was defined as a two-sided *p*-value < 0.05. All analyses were conducted using SAS version 9.4. Survey design, administration, and reporting were guided by the Consensus-Based Checklist for Reporting of Survey Studies (CROSS) (Supplemental file).^21^

## Results

### Respondent Characteristics

The overall survey response rate was 21% (208/1004), including 148 (72%) surgeons, 32 (16%) medical oncologists and 27 (13%) radiation oncologists (Table 1). Most respondents (73.6%) were from the USA, and 26.4% were from Canada. The earliest year respondents completed their terminal training was in 1977, and the most recent was 2024. The majority of the surgical (74%) and medical oncologists (65.6%) completed a breast-focused fellowship, and 52.6% of the respondents reported 100% of their clinical practice as breast.

**Table 1:** Characteristics of respondents and their clinical practice

Characteristics	*n*	%
*Specialty*^a^		
Surgeon	148	71.5
Radiation oncologist	27	13.04
Medical oncologist	32	15.46
*Years in practice*		
0–10	91	43.75
11–20	63	30.29
21–47	54	25.96
*Awareness, in years*		
0–5	151	72.6
5–10	45	21.63
11–13	12	5.77
*Region of practice*		
US Northeast	48	23.08
US Midwest	47	22.6
US South	35	16.83
US West	23	11.06
Canada	55	26.44
*Practice type*^a^		
Community hospital	39	18.84
Regional network	39	18.84
Academic medical center	126	60.87
Other	3	1.45
*Proportion of clinical practice on breast care*^a^		
100%	109	52.66
< 100%	98	47.34
*Average no. of new patients with breast cancer evaluated by participant each month*^a^		
> 35	14	6.8
31–35	7	3.4
26–30	17	8.2
21–25	32	15.5
16–20	47	22.7
11–15	44	21.3
5–10	34	16.4
< 5	12	5.8
*Primary practice has a breast surgical oncology fellowship*^b^		
Yes	68	46.3
No	79	53.7
*Completed breast or surgical oncology fellowship*^b^		
Yes	108	74.0
No	38	26.0
*Completed breast medical oncology fellowship*^c^		
Yes	21	65.6
No	11	34.4
*Frequency of clinical trial participation*^a^		
Not applicable	25	12.1
Never	5	2.4
Rarely	12	5.8
Sometimes	21	10.1
Often	76	36.7
Always	68	32.9

^a^Total does not add up to 208 (total respondents) due to missing responses

^b^Only surgeons were asked the question

^c^Only medical oncologists were asked the question

### SOUND Trial Awareness

The mean year respondents became aware of the SOUND trial was 2020 (range 2011–2024). The most common method by which respondents became aware of the SOUND trial was through direct communication with colleagues (*n* = 51 [24.5%]), followed by publication of the trial concept before the publication of the results (*n* = 37 [17.8%]) (Table 2). In total, 26 (12.5%) respondents became aware of the study through the publication of the results of the SOUND trial. Five respondents indicated that they became aware of the SOUND trial when invited to complete the survey.

**Table 2:** How participants first became aware of the SOUND clinical trial on omission of sentinel lymph node biopsy in early-stage breast cancer after negative axillary ultrasound

Method	*N* (*N* = 208)	%
Direct communication with colleague(s)	51	24.5
Publication of trial concept/design (before publication of results)	37	17.8
National conference	28	13.5
Publication of results (*JAMA Oncology* paper)	26	12.5
Local multidisciplinary conference (e.g. tumor board)	20	9.6
Other	14	6.7
During residency/fellowship training	9	4.3
Clinical trial cooperative group: NRG, Alliance, ECOG, SWOG, CCTG	8	3.8
Medical society bulletin board posting	5	2.4
Email blast from specialty organization	5	2.4
National guideline	3	1.4
Social media (e.g. Twitter/X, Instagram, etc.)	2	1.0

CCTG, Canadian Cancer Trials Group; ECOG, Eastern Cooperative Oncology Group; SWOG, Southwest Oncology Group

### Current Practice: Factors Associated with Currently Omitting SLNB

Respondents were asked whether they currently discuss SLNB omission as an option in select patients aged < 70 years with early-stage breast cancer; 99 (47.8%) responded yes and 108 (52.2%) responded no. In comparison, most (*N* = 200 [97%]) currently discuss SLNB omission as an option in patients aged ≥ 70 years. Overall, length of awareness of the SOUND trial was significantly associated with a higher likelihood of currently discussing SLNB omission in patients aged < 70 years (OR 1.13, *P* = 0.01) (Table 3). Physicians practicing in the Northeast USA were significantly more likely to discuss SLNB omission than were those in the Western USA (OR 3.45, *P* = 0.03). Years in practice, specialty type (including fellowship training), practice type, proportion of clinical practice in breast, whether respondents routinely discussed clinical trial enrollment with patients, and medical or radiation oncology referral workflow were not associated with current practice on SLNB omission.

**Table 3:** Factors associated with currently discussing omission of sentinel lymph node biopsy (SLNB) as an option in select patients aged < 70 years with early-stage breast cancer

Factor	OR (95% CI)	*P*-value
*Years in practice*		
0–10	0.81 (0.35–1.86)	0.623
11–20	0.50 (0.21–1.17)	0.11
21–47	*Ref*	
*Length of awareness of SOUND clinical trial*		
Continuous (year)	1.13 (1.02–1.25)	0.014
*Specialty*		
Surgeon completed fellowship	0.62 (0.22–1.78)	0.376
Surgeon did not complete fellowship (or unknown)	1.34 (0.34–5.26)	0.673
Medical oncologist	1.23 (0.37–4.09)	0.739
Radiation oncologist	*Ref*	
*Region of practice*		
Northeast	3.45 (1.11–10.69)	0.032
Midwest	1.78 (0.58–5.46)	0.314
South	1.94 (0.59–6.32)	0.274
West	*Ref*	
Canada	0.83 (0.21–3.29)	0.79
*Practice type*		
Academic medical center	1.62 (0.75–3.47)	0.218
Non-academic	*Ref*	
*Proportion of clinical practice on breast care*		
100%	1.67 (0.76–3.66)	0.204
< 100%	*Ref*	
*Discuss enrollment in the trial with patients*		
Often or always	1.11 (0.46–2.70)	0.814
Others	*Ref*	
*Workflow*		
Surgeon sees patient first. If operative case, surgeon operates then refers patient to medical or radiation oncologist.	0.97 (0.46–2.03)	0.939
Others	*Ref*	

CI, confidence interval; OR, odds ratio

### Future Practice: Factors Associated with Intent to Omit SLNB

Based on the MISII questions, most (N = 127 [61.6%]) planned to use the results of the SOUND trial to guide clinical decision-making, and this was a high priority for 116 (56%) of the respondents. This intent to omit did not significantly increase with length of awareness of the SOUND trial, years in practice, specialty, practice type, clinical trial enrollment behavior, or medical oncology/radiation oncology referral workflow (Supplemental Table 1). Respondents whose clinical practice was exclusively in breast care were more likely to implement the results from the SOUND trial (OR 2.22, *P* = 0.05), to consider using the results from the SOUND trial a high priority (OR 2.92, *P* = 0.008), and to plan to use all aspects of the SOUND trial in their clinical decision-making (OR 2.43, *P* = 0.03).

Among the 99 respondents who currently recommend SLNB omission in patients aged < 70 years, 63 had high intent to use SOUND trial results, 61 considered using SOUND trial results a high priority, and 52 considered using all aspects of SOUND. Among those already omitting SLNB, length of awareness of the SOUND trial was associated with higher intent to use the results from the SOUND trial (Supplemental Table 2). Each additional year of awareness of the SOUND trial was associated with a 14% increase in the intent to omit SLNB in patients aged <70 years (OR 1.14, *P* = 0.01) (Supplemental Table 2), compared with a 13% increase for all providers regardless of current use of SLNB in patients aged < 70 years (Table 3). Those with 100% of their clinical practice in breast were also more likely to use the results from the SOUND trial (OR 3.65, *P* = 0.006). Regional practice variation was also observed; physicians practicing in the Northeast USA and Midwest USA had a significantly higher intent to use the results from the SOUND trial than those in Western USA (OR 6.01, *P* = 0.01; OR 4.47, *P* = 0.03) but those in the Southern USA and Canada did not.

### Management of Axilla in Early-stage Breast Cancer

**Fig. 1:**
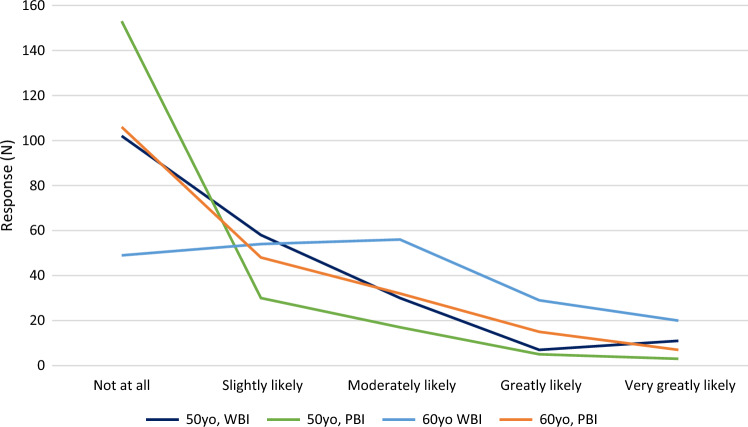
Participants were asked how likely they were to recommend omission of sentinel lymph node biopsy (SLNB) after a negative clinical exam and negative axillary ultrasound for the scenario of 1.5 cm grade 2 invasive ductal carcinoma undergoing lumpectomy. The scenarios varied by patient age (50 vs 60 years old [yo]) and radiation therapy (whole breast radiation [WBI] vs partial breast radiation [PBI])

Respondents were asked in a series of age-based (50 vs 60 years) and radiation therapy plan (whole breast radiation [WBI] vs partial breast radiation [PBI]) scenarios, their likelihood of recommending omission of SLNB (Fig. 1). All scenarios were based on a female patient with a 1.5 cm, grade 2, estrogen and progesterone receptor-positive, human epidermal growth factor receptor 2 (HER2)-negative invasive ductal carcinoma planning to undergo lumpectomy. In the scenario of a 60-year-old patient undergoing WBI, 105 respondents (50.5%) recommended omitting SLNB to a moderate extent or higher. In other scenarios, various factors were associated with a higher likelihood of omitting SLNB, yet none reached a majority consensus. Intent to omit SLNB was not affected by length of awareness of the SOUND trial, years in practice, specialty, proportion of clinical practice in breast, frequency of discussing clinical trial enrollment, or medical or radiation oncology referral workflow in any of the clinical scenarios.

In the clinical scenario of a 60-year-old patient undergoing WBI, region of practice was associated with omitting SLNB: those in the Southern USA were less likely to omit SLNB than those in the Western USA (OR 0.22, *P* = 0.02). Respondents in academic practice were more likely to consider omitting SLNB in patients aged 50 years planning to receive WBI (OR 3.1, *P* = 0.02) than were non-academic practice types. This difference by practice type was not observed for the other three scenarios. Medical oncologists were less likely than those in other specialties to consider omitting SLNB in patients aged 50 years undergoing WBI or aged 60 years undergoing WBI scenarios (OR 0.23, *P* = 0.049, OR 0.21, 0.014). This difference by provider specialty highlights the importance of multidisciplinary alignment in axillary management decision-making.

## Discussion

In this North American multidisciplinary clinician survey, we found variations in uptake of SLNB omission in patients aged < 70 years despite broad awareness of the SOUND clinical trial pre-dating the publication of the results. Awareness of the SOUND trial was largely spread through colleagues rather than formal publications. Although nearly all clinicians (> 97%) discuss omission of SLNB for patients aged ≥ 70 years, only about half (48%) do so for patients aged < 70 years. Longer awareness of the SOUND trial was associated with a greater likelihood of currently discussing omission of SLNB in patients aged < 70 years; among those already omitting SLNB, there was higher intent to use SOUND trial findings. Overall, 62% planned to use SOUND results and 56% viewed this as a high priority, with clinicians whose practice is exclusively breast care showing the strongest intent and prioritization.

Our study confirms the implementation challenges that were first demonstrated by the slow uptake of SLNB omission in patients aged ≥ 70 years. To overcome the multilevel barriers in older patients,^22^ previous studies have focused on conversation guides,^8,23–25^ decision aids,^26,27^ and electronic medical record prompts^28,29^ to change surgeon behavior. Given that early awareness of trial results may be associated with intent to change practice based on those trial results, especially among early adopters, leveraging these early adopters and their social networks may be an additional strategy that facilitates implementation. The potential for clinician networks and peer communication to drive change is supported by the social network interventions in implementation science.^30–33^ In our study, 24.5% of clinicians reported that they first learned about the SOUND clinical trial through direct communication with their colleagues, further supporting the potential for use of key early adopters and their social networks to increase SLNB omission.

We observed regional and specialty differences in SLNB omission recommendations, whereas other factors such as years in practice, fellowship training, practice type, and referral workflows did not affect the recommendation to omit SLNB. Regional variation in breast cancer surgery has been described previously, including differences in adoption of SLNB and axillary de‑escalation.^34–37^ These patterns likely reflect a combination of local practice norms, availability of multidisciplinary expertise, institutional culture, and payer or system‑level incentives, rather than purely patient‑level factors. From an implementation perspective, such geographic clustering suggests that strategies to promote de‑implementation of SLNB will need to be tailored to local contexts, engaging regional opinion leaders and addressing site‑specific barriers rather than assuming uniform uptake across North America.

In our study, earlier awareness of the SOUND trial was associated with current omission practices and with stronger intent among early adopters but not with hypothetical decisions across the entire cohort. This pattern raises questions about what “time of awareness” actually represents. One possibility is that earlier awareness is a marker of an underlying trait such as greater openness to implementation of new trial findings, higher engagement with new evidence, or stronger connections to academic networks. These characteristics may predispose some surgeons to adopt practice changes more readily than others. This pattern suggests that early awareness may preferentially influence a subset of clinicians who are already inclined toward de-implementation. Alternatively, the interval between awareness and practice change may be relatively constant across clinicians, and our cross‑sectional survey may simply be capturing a single point along a natural adoption curve. Under this scenario, a surgeon who first hears about the SOUND trial today might have the same intent to omit SLNB 2 years from now as a surgeon who first heard about the trial 2 years ago and is reporting their intent now.

An important question to consider is, “Is awareness of an ongoing trial inherently beneficial or can it be harmful if it prompts practice change before trial results are known?” In this study, the mean year of awareness of the SOUND trial was 2020, predating the publication of the primary results in 2023. When the eventual results of the SOUND trial and related clinical trials supported omission of SLNB, early awareness may have helped to shorten the lag between evidence generation and implementation. However, if trial outcomes had been negative or equivocal, such early shifts could have exposed patients to under-treatment. This dual possibility suggests that early dissemination should be accompanied by explicit messaging about the preliminary nature of the evidence, encouragement to consider changes within the context of clinical trials or multidisciplinary review, and rapid, clear communication once definitive results are available. Taken together, these considerations reinforce that successful and safe de-implementation of SLNB will require not only early and broad dissemination of trial findings but also structured, multidisciplinary processes to interpret emerging evidence, align radiation and systemic therapy planning, and calibrate practice change to the strength and maturity of the data.

This survey was not designed to evaluate real-world, multidisciplinary implementation of the SOUND trial findings. However, this is an important consideration for future studies designing implementation strategies when multiple disciplines are involved. Until recently, patients with early-stage breast cancer were mostly treated with lumpectomy and SLNB, followed by chemotherapy decision and WBI, with decision-making occurring in sequence. However, with the publication of several de-escalation clinical trials,^2,38–42^ there are potentially six different treatment options: SLNB/WBI, SLNB/PBI, SLNB/no radiation, no SLNB/WBI, no SLNB/PBI, or no SLNB/no radiation. Changing practice has been challenging, partly because of clinical trials testing de-escalation of different modalities one at a time. The SOUND clinical trial evaluated SLNB omission in patients with intent to receive WBI.^1^ Similarly, both INSEMA and BOOG 2013-08 required patients to undergo WBI.^2,43^ In contrast, radiation therapy studies evaluating PBI or no radiation required axillary staging, and medical oncology trials supporting chemotherapy omission only included patients who received standard axillary surgery and radiation therapy.^42,44^ Because even well-designed trials cannot address all potential clinically relevant nuances (e.g., variations and advancements in radiation therapy and systemic therapy), challenges in implementation will persist regardless of how long clinicians have been aware of the data. Thus, uptake is staggered as clinicians interpret and operationalize different trial results across the disciplines. Future work should therefore focus on understanding these multidisciplinary decision-making processes and on developing targeted implementation strategies to support consistent, evidence-aligned adoption of de-escalation of therapy in early-stage breast cancer.

This study has several limitations. First, the cross-sectional design precludes causal inference; we cannot determine whether earlier awareness caused changes in practice or whether clinicians inclined toward de-implementation sought out information about the SOUND trial earlier. Second, surgeons were overrepresented, raising the possibility of nonresponse bias and limiting generalizability to all clinicians involved in breast cancer care. Our sample also included a high proportion of clinicians with 100% of their practice in breast, which may overestimate both awareness of the SOUND trial and willingness to omit SLNB compared with the broader oncology community. As a result of purposive sampling, we acknowledge the responses skewed towards those whose practices were 100% in breast cancer. However, purposive sampling was chosen to ensure that the respondents had the relevant expertise for the survey questions. A larger survey study is currently in development, which will include a larger sample of community oncology practitioners. Third, all measures were self-reported and may be subject to recall and social desirability bias, particularly regarding current omission practices. However, we employed neutral and non-judgmental phrasing in survey questions to mitigate this effect. Finally, scenario-based questions cannot fully capture the complexity of real-world decision-making, including patient preferences, comorbidities, and local resource constraints.

## Conclusion

Our study found that, although awareness and intent to use the SOUND clinical trial are present, actual practice change is variable and context dependent. Early awareness of the SOUND trial is associated with actual current practice and intent among early adopters but was not associated with hypothetical scenario-based decisions across the whole cohort. Based on factors associated with current practice or intent, our study underscores that awareness, specialty norms, and regional practice culture rather than clinician tenure or training background may be driving SLNB omission based on new clinical trial results. Future studies should develop and test coordinated multidisciplinary strategies to de-implement SLNB that address specialty-specific needs (including impact of SLNB omission on radiation and systemic therapy planning), and regional practice culture, supported by clear guidelines and robust dissemination through clinician networks.

## Supplementary Information

Below is the link to the electronic supplementary material.

Supplementary file1 (DOCX 28 KB)

Supplementary file2 (DOCX 21 KB)

## Data Availability

The datasets generated during and/or analyzed during the current study are available from the corresponding author on reasonable request.

## Prior presentation

Presented in part as a poster at the 2025 San Antonio Breast Cancer Symposium.
