# Supplementary material for: From Clinical Trial Awareness to Practice: Factors Associated with Intent to Omit Axillary Surgery Based on the SOUND Clinical Trial
Source: Ann Surg Oncol. 2026 May 14;33(9):8165–74. doi: 10.1245/s10434-026-19792-x (PMC13452869; doi:10.1245/s10434-026-19792-x)
Supplement: Supplementary file 1 [file 10434_2026_19792_MOESM1_ESM.docx]

**Supplemental file**

**From Clinical Trial Awareness to Practice: Factors Associated with Intent to Omit Axillary Surgery Based on the SOUND Clinical Trial**

Supplemental Tables: 2

Supplemental Table 1. Factors associated with intent to omit SLNB (based on MISII questions) for all participants. MISII 1) I use/ plan to use the results from the SOUND clinical trial to guide my clinical decision-making; MISII 2) Using the results from the SOUND clinical trial into my clinical decision-making is a high priority for me; MISII 3) I use/ will use all aspects of SOUND clinical trial results into my clinical decision-making with my patients.

|  | **MISII 1** | | **MISII 2** | | **MISII 3** | | |
| --- | --- | --- | --- | --- | --- | --- | --- |
|  | **OR (95% CI)** | **P-value** | **OR (95% CI)** | **P-value** | **OR (95% CI)** | **P-value** |  |
| **Years in Practice** |  |  |  |  |  |  |  |
| 0-10 | 1.63 (0.72, 3.69) | 0.241 | 1.52 (0.67, 3.42) | 0.314 | 1.65 (0.75, 3.61) | 0.213 |  |
| 11-20 | 1.29 (0.56, 2.93) | 0.549 | 0.99 (0.44, 2.23) | 0.978 | 1.44 (0.65, 3.17) | 0.371 |  |
| 21-47 | *Ref* |  | *Ref* |  | *Ref* |  |  |
| **Length of awareness of SOUND clinical trial** |  |  |  |  |  |  |  |
| Continuous (year) | 1.08 (0.98, 1.19) | 0.105 | 1.09 (0.99, 1.19) | 0.075 | 1.02 (0.94, 1.12) | 0.63 |  |
| **Specialty** |  |  |  |  |  |  |  |
| Surgeon completed fellowship | 0.67 (0.24, 1.90) | 0.452 | 1.11 (0.39, 3.13) | 0.847 | 0.58 (0.21, 1.60) | 0.293 |  |
| Surgeon did not complete  fellowship (or unknown) | 1.81 (0.47, 6.94) | 0.387 | 3.33 (0.89, 12.47) | 0.074 | 0.78 (0.22, 2.77) | 0.696 |  |
| Medical Oncologist | 0.63 (0.19, 2.06) | 0.445 | 1.43 (0.44, 4.67) | 0.551 | 0.62 (0.19, 2.01) | 0.431 |  |
| Radiation Oncologist | *Ref* |  | *Ref* |  | *Ref* |  |  |
| **Region of Practice** |  |  |  |  |  |  |  |
| Northeast | 1.99 (0.65, 6.06) | 0.227 | 1.31 (0.43, 4.04) | 0.636 | 0.72 (0.24, 2.14) | 0.562 |  |
| Midwest | 1.81 (0.59, 5.56) | 0.3 | 1.06 (0.34, 3.29) | 0.924 | 1.45 (0.48, 4.37) | 0.51 |  |
| South | 1.34 (0.41, 4.34) | 0.628 | 1.04 (0.32, 3.44) | 0.947 | 0.80 (0.25, 2.56) | 0.709 |  |
| West | *Ref* |  | *Ref* |  | *Ref* |  |  |
| Canada | 5.08 (1.27, 20.28) | 0.021 | 2.95 (0.75, 11.63) | 0.122 | 2.73 (0.72, 10.36) | 0.14 |  |
| **Practice Type** |  |  |  |  |  |  |  |
| Academic Medical Center | 0.58 (0.27, 1.26) | 0.172 | 0.81 (0.38, 1.70) | 0.572 | 0.86 (0.41, 1.78) | 0.68 |  |
| Non-Academic | *Ref* |  | *Ref* |  | *Ref* |  |  |
| **Proportion of clinical practice on breast care** |  |  |  |  |  |  |  |
| 100% | 2.22 (1.00, 4.94) | 0.05 | 2.92 (1.32, 6.46) | 0.008 | 2.43 (1.10, 5.33) | 0.027 |  |
| <100% | *Ref* |  | *Ref* |  | *Ref* |  |  |
| **Discuss enrollment in the trial with patients** |  |  |  |  |  |  |  |
| Often or always | 2.30 (0.95, 5.61) | 0.066 | 1.48 (0.62, 3.52) | 0.382 | 1.15 (0.49, 2.69) | 0.743 |  |
| Others | *Ref* |  | *Ref* |  | *Ref* |  |  |
| **Work flow** |  |  |  |  |  |  |  |
| Surgeon sees patient first. If  operative case, surgeon operates  then refers patient to medical or  radiation oncologist. | 0.56 (0.27, 1.18) | 0.127 | 0.66 (0.32, 1.37) | 0.265 | 0.44 (0.21, 0.90) | 0.025 |  |
| Others | *Ref* |  | *Ref* |  | *Ref* |  |  |

Supplemental Table 2. Factors associated with intent to omit SLNB (based on MISII questions) among participants who currently discuss SLNB omission as an option in patients < 70 years old. MISII 1) I use/ plan to use the results from the SOUND clinical trial to guide my clinical decision-making; MISII 2) Using the results from the SOUND clinical trial into my clinical decision-making is a high priority for me; MISII 3) I use/ will use all aspects of SOUND clinical trial results into my clinical decision-making with my patients.

|  | **MISII 1** | | **MISII 2** | | **MISII 3** | |
| --- | --- | --- | --- | --- | --- | --- |
|  | **OR (95% CI)** | **P-value** | **OR (95% CI)** | **P-value** | **OR (95% CI)** | **P-value** |
| **Years in Practice** |  |  |  |  |  |  |
| 0-10 | 0.94 (0.38, 2.33) | 0.894 | 1.16 (0.46, 2.93) | 0.761 | 1.03 (0.41, 2.61) | 0.947 |
| 11-20 | 0.34 (0.13, 0.91) | **0.032** | 0.40 (0.15, 1.09) | 0.073 | 0.61 (0.23, 1.61) | 0.317 |
| 21-47 | *Ref* |  | *Ref* |  | *Ref* |  |
| **Length of awareness of SOUND clinical trial** |  |  |  |  |  |  |
| Continuous (year) | 1.14 (1.03, 1.26) | **0.012** | 1.12 (1.01, 1.25) | **0.025** | 1.06 (0.96, 1.18) | 0.212 |
| **Specialty** |  |  |  |  |  |  |
| Surgeon completed fellowship | 0.60 (0.17, 2.08) | 0.421 | 0.74 (0.20, 2.73) | 0.657 | 0.56 (0.17, 1.83) | 0.338 |
| Surgeon did not complete  fellowship (or unknown) | 1.51 (0.32, 7.07) | 0.602 | 2.32 (0.48, 11.18) | 0.293 | 0.94 (0.21, 4.19) | 0.934 |
| Medical Oncologist | 0.66 (0.17, 2.60) | 0.555 | 1.05 (0.25, 4.38) | 0.943 | 0.73 (0.20, 2.74) | 0.647 |
| Radiation Oncologist | *Ref* |  | *Ref* |  | *Ref* |  |
| **Region of Practice** |  |  |  |  |  |  |
| Northeast | 6.01 (1.55, 23.36) | **0.01** | 2.60 (0.74, 9.07) | 0.135 | 2.31 (0.62, 8.58) | 0.213 |
| Midwest | 4.47 (1.12, 17.95) | **0.034** | 1.64 (0.45, 6.03) | 0.453 | 2.63 (0.69, 10.10) | 0.159 |
| South | 2.93 (0.68, 12.70) | 0.15 | 1.64 (0.42, 6.39) | 0.475 | 2.14 (0.52, 8.74) | 0.291 |
| West | *Ref* |  | *Ref* |  | *Ref* |  |
| Canada | 3.50 (0.64, 19.04) | 0.148 | 2.00 (0.40, 10.02) | 0.401 | 2.46 (0.47, 12.96) | 0.29 |
| **Practice Type** |  |  |  |  |  |  |
| Academic Medical Center | 0.85 (0.36, 1.98) | 0.707 | 1.23 (0.52, 2.91) | 0.637 | 1.40 (0.59, 3.32) | 0.451 |
| Non-Academic | *Ref* |  | *Ref* |  | *Ref* |  |
| **Proportion of clinical practice on breast care** |  |  |  |  |  |  |
| 100% | 3.65 (1.45, 9.21) | **0.006** | 5.10 (1.96, 13.31) | **0.001** | 3.01 (1.21, 7.50) | **0.018** |
| <100% | *Ref* |  | *Ref* |  | *Ref* |  |
| **Discuss enrollment in the trial with patients** |  |  |  |  |  |  |
| Often or always | 2.47 (0.90, 6.78) | 0.08 | 1.64 (0.60, 4.49) | 0.334 | 1.50 (0.54, 4.16) | 0.434 |
| Others | *Ref* |  | *Ref* |  | *Ref* |  |
| **Work flow** |  |  |  |  |  |  |
| Surgeon sees patient first. If  operative case, surgeon operates  then refers patient to medical or  radiation oncologist. | 0.86 (0.39, 1.88) | 0.705 | 0.74 (0.34, 1.63) | 0.458 | 0.63 (0.29, 1.38) | 0.249 |
| Others | *Ref* |  | *Ref* |  | *Ref* |  |
